# CBPR: AN EFFECTIVE APPROACH TO RECRUIT AND RETAIN MINORITY OLDER REFUGEE ADULTS IN COMMUNITY-BASED RESEARCH

**DOI:** 10.1093/geroni/igac059.638

**Published:** 2022-12-20

**Authors:** Sudha Shreeniwas

**Affiliations:** UNC Greensboro, Greensboro, North Carolina, United States

## Abstract

Community based participatory research (CBPR) builds mutually beneficial partnerships between diverse communities and researchers, yielding improved communication among stakeholders, more interest in research participation among community older adults, and improved insights on health disparities. CBPR approaches have great utility for culturally appropriate research design, data collection and analyses, and dissemination with minority underserved communities. We describe outreach, research methods, and community capacity-building from three projects with refugee communities in Greensboro, NC: the Montagnard Hypertension Research Project; The Refugee Older Adults Needs Assessment Project; and the Elder Abuse and Neglect exploratory study. These projects were initiated by community leaders, with technical partnership provided by university researchers and students. We used community-centric methods, and collected a range of data including interviews, photographs, oral histories, surveys, and biological specimens. These efforts have advanced social justice and health equity agendas for both academic and community partners, working to advance health of older adults.

